# Crystal structure of bis­(1-ethyl-1*H*-imidazole-κ*N* ^3^)(5,10,15,20-tetra­phenyl­porphyrinato-κ^4^ *N*)iron(II) tetra­hydro­furan monosolvate

**DOI:** 10.1107/S2056989018006308

**Published:** 2018-05-01

**Authors:** Wei Ding, Jianfeng Li

**Affiliations:** a College of Materials Science and Optoelectronic Technology, University of Chinese Academy of Sciences, Yianqi Lake, Huairou District, Beijing 101408, People’s Republic of China

**Keywords:** crystal structure, C—H⋯π inter­action, 1-ethyl­imidazole, iron(II)

## Abstract

The title complex, [Fe(C_44_H_28_N_4_)(C_5_H_8_N_2_)_2_]·C_4_H_8_O, possesses inversion symmetry with the iron(II) atom located on a center of symmetry. The metal atom is coordinated in a symmetric octa­hedral geometry by four pyrrole N atoms of the porphyrin ligand in the equatorial plane and two N atoms of 1-ethyl­imidazole ligands in the axial sites; the complex crystallizes with a tetra­hydro­furan solvent mol­ecule. The average Fe—N_p_ (N_p_ is a porphyrin N atom) bond length is 1.995 (3) Å and the axial Fe—N_Im_ (N_Im_ is an imidazole N atom) bond length is 1.994 (2) Å. The two 1-ethyl­imidazole ligands are mutually parallel. The dihedral angle between the 1-ethyl­imidazole plane and the plane of the closest Fe—N_p_ vector is 24.5°. In the crystal, the only significant inter­molecular inter­actions present are C—H⋯π inter­actions.

## Abstract 2

The title iron(II)–porphyrin complex possesses inversion symmetry with the metal atom located on a center of symmetry. The iron(II) atom is coordinated in a symmetric octa­hedral geometry by four pyrrole N atoms of the porphyrin ligand in the equatorial plane and two N atoms of 1-ethyl­imidazole ligands in the axial sites. The dihedral angle between the 1-ethyl­imidazole plane and the plane of the closest Fe—N_p_ vector is 24.5(**?**)°.

## Chemical context   

Bis-histidine coordinated hemes are present in various cytochrome *b* complexes, and are known to be involved in electron-transfer processes (Xia *et al.*, 1997 ▸). As models of these six-coordinate heme complexes, a number of single-crystal structures of [Fe(II,III)(Porph)(*L*)_2_]**^0,+^** (Porph is a porphyrinato ligand and *L* is a N-donor imidazole ligand) have been reported (Walker, 2004 ▸). The first ferrous porphyrin crystal structure with two 1-ethyl­imidazole ligands is [Fe^II^(TpivPP)(1-EtIm)_2_]·0.5C_7_H_8_ [TpivPP = *α,α,α,α*-tetra­kis­(*o*-pivalamido­phen­yl)porphyrinato; 1-EtIm = 1-ethyl­imidazole], which was reported by Li and co-workers (Li *et al.*, 2008 ▸). Later, another analogue of [Fe^II^(TFPPBr_8_)(1-EtIm)_2_] [TFPPBr_8_ = 2,3,7,8,12,13,17,18-octa­bromo-5,10,15,20-tetra­kis­(penta­fluoro­phen­yl)πorphyrinato] was reported (Hu *et al.*, 2016 ▸). Herein, we report the structural properties of the iron(II)–porphyrin complex [Fe^II^(TPP)(1-EtIm)_2_]·THF where the metal center is likewise octa­hedrally coordinated.

## Structural commentary   

**Figure 1:**
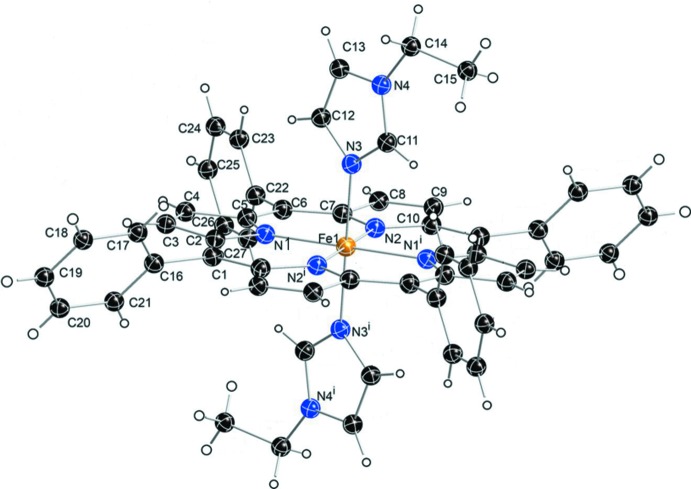
The mol­ecular structure of the title complex, with displacement ellipsoids drawn at the 50% probability level. The disordered THF mol­ecule has been omitted for clarity, and unlabelled atoms are related to labelled atoms by the inversion symmetry code: (i) −*x*, −*y* + 1, −*z* + 1.

**Figure 2:**
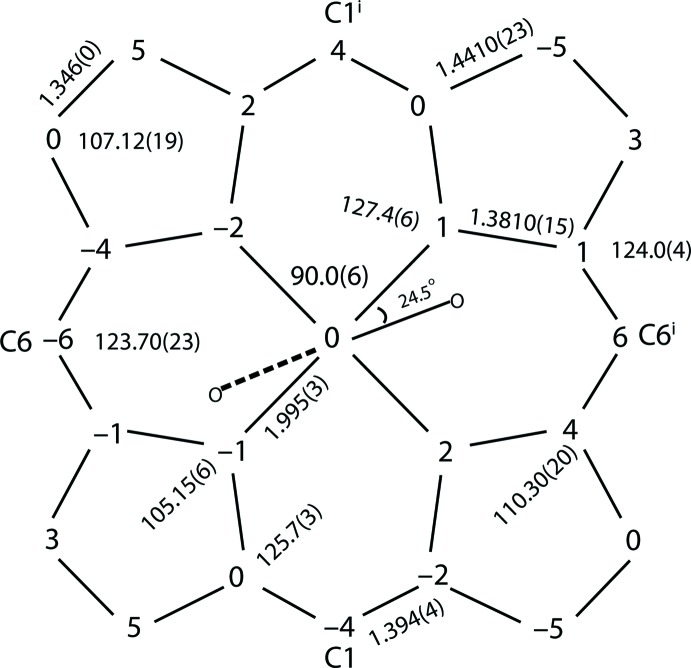
Formal diagram of the porphyrinate core of [Fe^II^(TPP)(1-EtIm)_2_]. Averaged values of the chemically unique bond distances (in Å) and angles (in °) are shown. The numbers in parentheses are the e.s.d.’s calculated on the assumption that the averaged values were all drawn from the same population. The perpendicular displacements (in units of 0.01 Å) of the porphyrin core atoms from the 24-atom mean plane are also displayed. Positive values of the displacement are towards the hindered porphyrin side, the solid line and dashed line indicate the plane of imidazole on the unhindered porphyrin side.

The asymmetric unit of the title compound (Fig. 1 ▸), contains half of an Fe^II^ porphyrin complex, with the iron(II) atom located on an inversion center, an 1-ethyl­imidazole ligand mol­ecule, and half of a THF solvent mol­ecule. The THF mol­ecule is disordered over two positions**;** the site occupancy factors (SOFs) of the two disordered moieties being 0.35 and 0.15. The two 1-ethyl­imidazole ligands of [Fe^II^(TPP)(1-EtIm)_2_] are mutually parallel, as required by the crystal symmetry. Additional qu­anti­tative information about the structure is displayed in Fig. 2 ▸, which includes the displacement of each porphyrin core atom (in units of 0.01 Å) from the 24-atom mean plane. The orientation of the 1-ethyl­imidazole ligand including the value of the dihedral angles is also given. As can be seen in Fig. 2 ▸, the porphyrin core of [Fe^II^(TPP)(1-EtIm)_2_] is near-planar and the iron(II) atom is in the 24-atom plane. The displacements of every porphyrin core atom is less than 0.06 Å.
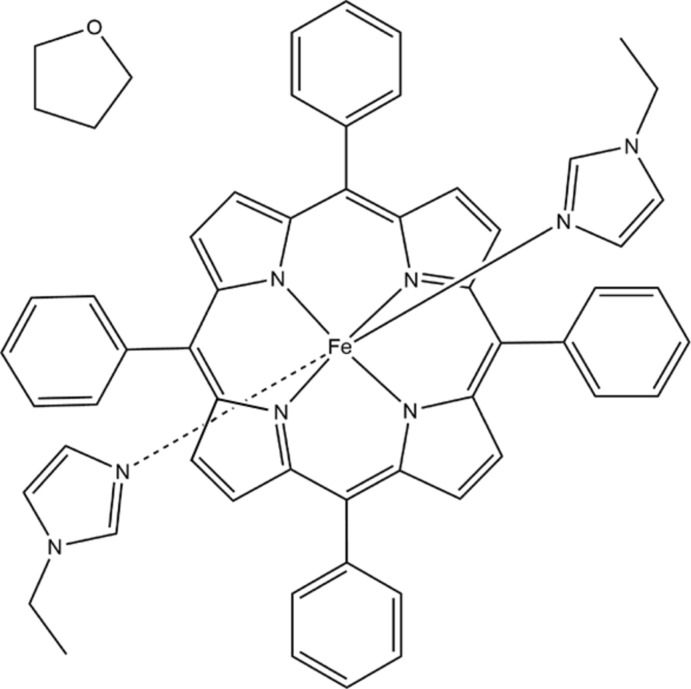


The average Fe—N_p_ bond length of 1.995 (3) Å is similar to 1.993 (6) Å in [Fe^II^(TpivPP)(1-EtIm)_2_] (Li *et al.*, 2008 ▸) and 1.994 (10) Å in [Fe^II^(TFPPBr_8_)(1-EtIm)_2_] (Hu *et al.*, 2016 ▸), which are typical values for six-coordinated low-spin (porphinato)iron(II) derivatives (Scheidt *et al.*, 1981 ▸). The axial Fe—N_Im_ bond length is 1.994 (2) Å. The average N_p_—Fe—N_p_ angle is ideal at 90.00 (6)°. The dihedral angle between the 1-ethyl­imidazole plane and the plane of the closest Fe—N_p_ vector is 24.5°.

## Supra­molecular features   

**Figure 3:**
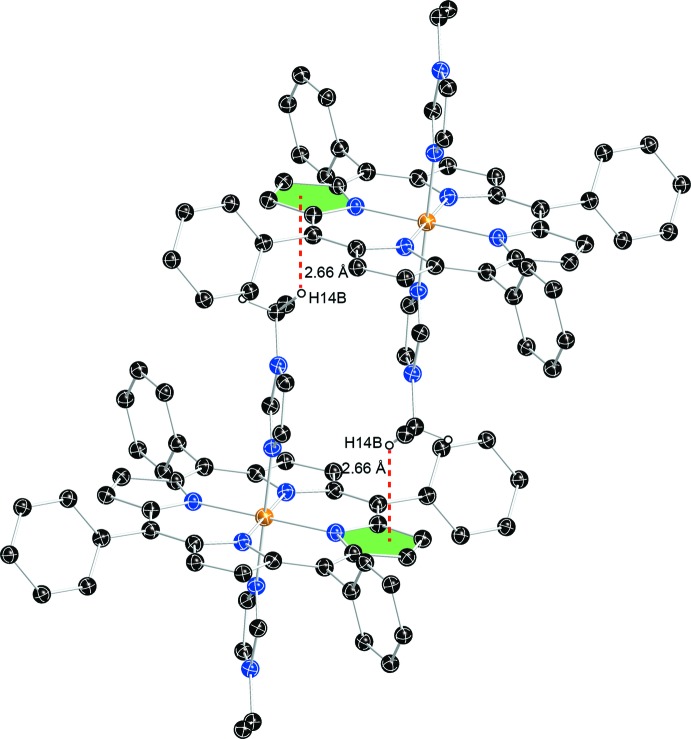
The C—H⋯π inter­actions in the title compound. Dashed lines show the distances between H atoms of 1-ethyl­imidazole and the pyrrole core planes. Solvent (THF) mol­ecules and other H atoms have been omitted for clarity.

**Figure 4:**
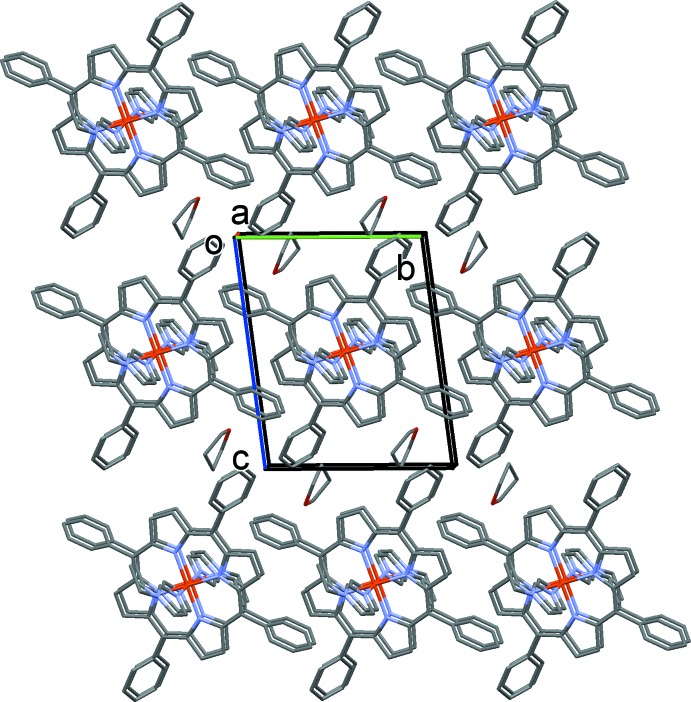
A view along the *a* axis of the mol­ecular packing of the title compound. H atoms have been omitted for clarity.

In the title compound, as shown in Fig. 3 ▸, the distance between the hydrogen atom H14*B* (C14) of the ethyl group of 1-EtIm and the pyrrole plane of the neighboring porphyrin is 2.66 Å, smaller than 2.9 Å, which is a limit suggested for the existence of a C—H⋯π inter­action (Takahashi *et al.*, 2001 ▸). Details of this inter­action are given in Table 1 ▸. The mol­ecular packing is shown in Fig. 4 ▸.

## Synthesis and crystallization   

**4.1 General information**. All reactions were done using standard Schlenk techniques unless otherwise specified. All solvents were freeze/pump/thaw/degassed prior to use. Benzene and tetra­hydro­furan (THF) were refluxed in the presence of sodium and benzo­phenone under argon until the solution was blue. Hexanes was distilled from sodium/potassium alloy under argon. Ethane­thiol and 1-ethyl­imidazole were distilled under an argon atmosphere. (H_2_TPP), [Fe(TPP)Cl] and [Fe(TPP)]_2_O were prepared according to the literature method (Adler *et al.*, 1967 ▸, 1970 ▸; Fleischer & Srivastava, 1969 ▸).

**4.2 Synthesis of bis­(1-ethyl-1** ***H*** **-imidazole-κ** ***N*** **^3^)(5,10,15,20-tetra­phenyl­porphyrinato-κ^4^** ***N*** **)iron(II) tetra­hydro­furan monosolvate**

The purple powder [Fe(TPP)]_2_O (15.9 mg, 0.018 mmol) was dried in vacuum for 1 h in a Schlenk tube. Benzene (∼5 ml) was transferred into the Schlenk tube by cannula and ethane­thiol (2 ml, 0.028 mol) was added *via* syringe. The mixture was stirred under argon at ambient temperature. After 36 h, the reduction was complete and the solvent was evaporated by pump. THF (∼5 ml) was transferred into the Schlenk tube *via* a cannula, and 1-ethyl­imidazole (0.5 ml, 5.19 mmol) was added using a syringe. Hexanes were then allowed to diffuse slowly into the reaction solution. After several weeks purple block-shaped crystals of the title compound were obtained.

## Refinement   

Crystal data, data collection and structure refinement details are summarized in Table 2 ▸. The hydrogen atoms (H14*A*, H14*B*) attached to atom C14 of the 1-ethyl­imidazole ligand were located in a difference-Fourier map and refined freely. All other hydrogen atoms were placed in calculated positions (C—H = 0.95, 0.98 and 0.99 Å for aryl, methyl and methlyene H atoms, respectively) and refined using a riding model with *U*_iso_(H) = 1.5*U*_eq_(C) for methyl H atoms or *U*_iso_(H) = 1.2U_eq_(C) otherwise. The C—O, C—C, C⋯C distances in the disordered THF mol­ecule were constrained to 1.42 (1), 1.50 (1) and 1.55 (1) Å, respectively. Six atoms (C30*A*, C28*B*, C29*B*, C30*B*, C31*B*, O1*B*) of the THF solvent mol­ecule exhibited unusual thermal motion and were restrained by a SIMU command. Five outlier reflections were omitted in the final cycles of refinement.

## Supplementary Material

Crystal structure: contains datablock(s) I. DOI: 10.1107/S2056989018006308/qm2123sup1.cif

Structure factors: contains datablock(s) I. DOI: 10.1107/S2056989018006308/qm2123Isup2.hkl

CCDC reference: 1839313

Additional supporting information:  crystallographic information; 3D view; checkCIF report

## Figures and Tables

**Table 1:** Hydrogen-bond geometry (Å, °) *Cg* is the centroid of the N2/C7–C10 ring.

*D*—H⋯*A*	*D*—H	H⋯*A*	*D*⋯*A*	*D*—H⋯*A*
C14—H14*B*⋯*Cg* ^i^	0.99 (4)	2.69 (4)	3.437 (3)	133 (2)

Symmetry code: (i) 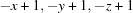.

**Table 2:** Experimental details

Crystal data	
Chemical formula	[Fe(C_44_H_28_N_4_)(C_5_H_8_N_2_)_2_]·C_4_H_8_O
*M*_r_	932.92
Crystal system, space group	Triclinic, *P* 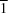
Temperature (K)	100
*a*, *b*, *c* (Å)	9.2962 (3), 10.7051 (4), 13.4920 (5)
α, β, γ (°)	79.809 (1), 76.034 (1), 75.933 (1)
*V* (Å^3^)	1253.90 (8)
*Z*	1
Radiation type	Mo *K*α
μ (mm^−1^)	0.35
Crystal size (mm)	0.26 × 0.17 × 0.08

Data collection	
Diffractometer	Bruker D8 QUEST System
Absorption correction	Multi-scan (*SADABS*; Bruker, 2014 ▸)
*T*_min_, *T*_max_	0.931, 0.972
No. of measured, independent and observed [*I* > 2σ(*I*)] reflections	19292, 5147, 4542
*R*_int_	0.044
(sin θ/λ)_max_ (Å^−1^)	0.626

Refinement	
*R*[*F* ^2^ > 2σ(*F* ^2^)], *wR*(*F* ^2^), *S*	0.058, 0.167, 1.11
No. of reflections	5147
No. of parameters	386
No. of restraints	113
H-atom treatment	H atoms treated by a mixture of independent and constrained refinement
Δρ_max_, Δρ_min_ (e Å^−3^)	1.53, −0.47

Computer programs: *APEX2* (Bruker, 2014 ▸), *SHELXL2014* (Sheldrick, 2015*b* ▸), *SAINT-Plus* (Bruker, 2014 ▸) and *XPREP* (Bruker, 2014 ▸), *SHELXT* (Sheldrick, 2015*a* ▸), *XP* (Sheldrick, 2008 ▸) and *Mercury* (Macrae *et al.*, 2008 ▸), *XCIF* (Sheldrick, 2008 ▸) and *enCIFer* (Allen *et al.*, 2004 ▸).

## supplementary crystallographic information

### Crystal data

**Table:**

[Fe(C_44_H_28_N_4_)(C_5_H_8_N_2_)_2_]·C_4_H_8_O	*Z* = 1
*M**_r_* = 932.92	*F*(000) = 490
Triclinic, *P*1	*D*_x_ = 1.235 Mg m^−^^3^
*a* = 9.2962 (3) Å	Mo *K*α radiation, λ = 0.71073 Å
*b* = 10.7051 (4) Å	Cell parameters from 9016 reflections
*c* = 13.4920 (5) Å	θ = 2.4–26.4°
α = 79.809 (1)°	µ = 0.35 mm^−^^1^
β = 76.034 (1)°	*T* = 100 K
γ = 75.933 (1)°	Block, purple
*V* = 1253.90 (8) Å^3^	0.26 × 0.17 × 0.08 mm

### Data collection

**Table:**

Bruker D8 QUEST System diffractometer	4542 reflections with *I* > 2σ(*I*)
Radiation source: fine-focus sealed tube	*R*_int_ = 0.044
φ and ω scans	θ_max_ = 26.4°, θ_min_ = 2.5°
Absorption correction: multi-scan (SADABS; Bruker, 2014)	*h* = −11→11
*T*_min_ = 0.931, *T*_max_ = 0.972	*k* = −13→13
19292 measured reflections	*l* = −16→16
5147 independent reflections	

### Refinement

**Table:**

Refinement on *F*^2^	113 restraints
Least-squares matrix: full	Hydrogen site location: mixed
*R*[*F*^2^ > 2σ(*F*^2^)] = 0.058	H atoms treated by a mixture of independent and constrained refinement
*wR*(*F*^2^) = 0.167	*w* = 1/[σ^2^(*F*_o_^2^) + (0.0784*P*)^2^ + 2.6414*P*] where *P* = (*F*_o_^2^ + 2*F*_c_^2^)/3
*S* = 1.11	(Δ/σ)_max_ < 0.001
5147 reflections	Δρ_max_ = 1.53 e Å^−^^3^
386 parameters	Δρ_min_ = −0.46 e Å^−^^3^

### Special details

**Table:**

Geometry. All esds (except the esd in the dihedral angle between two l.s. planes) are estimated using the full covariance matrix. The cell esds are taken into account individually in the estimation of esds in distances, angles and torsion angles; correlations between esds in cell parameters are only used when they are defined by crystal symmetry. An approximate (isotropic) treatment of cell esds is used for estimating esds involving l.s. planes.
Refinement. Refinement of F^2^ against ALL reflections. The weighted R-factor wR and goodness of fit S are based on F^2^, conventional R-factors R are based on F, with F set to zero for negative F^2^. The threshold expression of F^2^ > 2sigma(F^2^) is used only for calculating R-factors(gt) etc. and is not relevant to the choice of reflections for refinement. R-factors based on F^2^ are statistically about twice as large as those based on F, and R- factors based on ALL data will be even larger.

### Fractional atomic coordinates and isotropic or equivalent isotropic displacement parameters (Å^2^)

**Table:**

	*x*	*y*	*z*	*U*_iso_*/*U*_eq_	Occ. (<1)
Fe1	0.0000	0.5000	0.5000	0.00924 (16)	
N3	0.1978 (2)	0.5561 (2)	0.45844 (17)	0.0121 (4)	
N4	0.3804 (2)	0.6544 (2)	0.45874 (17)	0.0138 (5)	
N1	0.0234 (2)	0.4581 (2)	0.35771 (16)	0.0118 (4)	
N2	0.1056 (2)	0.3198 (2)	0.54207 (16)	0.0108 (4)	
C1	−0.1057 (3)	0.6676 (3)	0.2771 (2)	0.0130 (5)	
C2	−0.0246 (3)	0.5395 (3)	0.2750 (2)	0.0141 (5)	
C3	0.0205 (3)	0.4727 (3)	0.1849 (2)	0.0205 (6)	
H3	0.0037	0.5084	0.1180	0.025*	
C4	0.0910 (3)	0.3506 (3)	0.2132 (2)	0.0196 (6)	
H4	0.1318	0.2835	0.1704	0.024*	
C5	0.0927 (3)	0.3410 (3)	0.3208 (2)	0.0137 (5)	
C6	0.1548 (3)	0.2286 (2)	0.3780 (2)	0.0127 (5)	
C7	0.1616 (3)	0.2208 (2)	0.4811 (2)	0.0120 (5)	
C8	0.2351 (3)	0.1062 (3)	0.5377 (2)	0.0147 (5)	
H8	0.2822	0.0254	0.5126	0.018*	
C9	0.2248 (3)	0.1346 (3)	0.6327 (2)	0.0144 (5)	
H9	0.2643	0.0783	0.6871	0.017*	
C10	0.1421 (3)	0.2673 (2)	0.6363 (2)	0.0114 (5)	
C11	0.2390 (3)	0.6380 (3)	0.5032 (2)	0.0152 (5)	
H11	0.1767	0.6800	0.5598	0.018*	
C12	0.3189 (3)	0.5190 (3)	0.3805 (2)	0.0168 (5)	
H12	0.3230	0.4604	0.3340	0.020*	
C13	0.4321 (3)	0.5794 (3)	0.3804 (2)	0.0183 (6)	
H13	0.5283	0.5710	0.3346	0.022*	
C14	0.4615 (3)	0.7433 (3)	0.4831 (2)	0.0193 (6)	
H14A	0.454 (4)	0.818 (3)	0.431 (3)	0.021 (8)*	
H14B	0.570 (4)	0.701 (3)	0.468 (3)	0.024 (9)*	
C15	0.4039 (3)	0.7774 (3)	0.5914 (2)	0.0204 (6)	
H15A	0.2967	0.8210	0.6004	0.031*	
H15B	0.4627	0.8354	0.6041	0.031*	
H15C	0.4148	0.6980	0.6403	0.031*	
C16	−0.1574 (3)	0.7373 (3)	0.1816 (2)	0.0153 (5)	
C17	−0.1145 (4)	0.8526 (3)	0.1325 (2)	0.0235 (6)	
H17	−0.0500	0.8880	0.1596	0.028*	
C18	−0.1651 (4)	0.9162 (3)	0.0440 (2)	0.0304 (7)	
H18	−0.1365	0.9956	0.0122	0.037*	
C19	−0.2562 (4)	0.8655 (3)	0.0023 (2)	0.0307 (8)	
H19	−0.2888	0.9087	−0.0588	0.037*	
C20	−0.2998 (4)	0.7512 (3)	0.0498 (2)	0.0265 (7)	
H20	−0.3630	0.7159	0.0214	0.032*	
C21	−0.2516 (3)	0.6879 (3)	0.1388 (2)	0.0194 (6)	
H21	−0.2830	0.6098	0.1711	0.023*	
C22	0.2182 (3)	0.1072 (3)	0.3273 (2)	0.0138 (5)	
C23	0.3640 (3)	0.0857 (3)	0.2667 (2)	0.0156 (5)	
H23	0.4234	0.1495	0.2557	0.019*	
C24	0.4230 (3)	−0.0285 (3)	0.2222 (2)	0.0195 (6)	
H24	0.5230	−0.0426	0.1815	0.023*	
C25	0.3368 (3)	−0.1223 (3)	0.2370 (2)	0.0210 (6)	
H25	0.3771	−0.1998	0.2059	0.025*	
C26	0.1915 (4)	−0.1018 (3)	0.2975 (2)	0.0230 (6)	
H26	0.1324	−0.1659	0.3084	0.028*	
C27	0.1325 (3)	0.0123 (3)	0.3422 (2)	0.0201 (6)	
H27	0.0329	0.0259	0.3834	0.024*	
O1A	0.6922 (6)	0.1716 (5)	0.1646 (4)	0.0128 (6)	0.35
C29A	0.8416 (9)	0.3157 (7)	0.0625 (5)	0.0124 (7)	0.35
H29A	0.9472	0.3111	0.0233	0.015*	0.35
H29B	0.8013	0.4026	0.0856	0.015*	0.35
C28A	0.8331 (9)	0.2105 (7)	0.1517 (5)	0.0127 (7)	0.35
H28A	0.9189	0.1358	0.1379	0.015*	0.35
H28B	0.8380	0.2429	0.2148	0.015*	0.35
C30A	0.7387 (8)	0.2857 (7)	−0.0043 (5)	0.0122 (7)	0.35
H30A	0.6901	0.3657	−0.0431	0.015*	0.35
H30B	0.7972	0.2230	−0.0528	0.015*	0.35
C31A	0.6257 (9)	0.2286 (7)	0.0775 (5)	0.0128 (7)	0.35
H31A	0.5353	0.2970	0.0981	0.015*	0.35
H31B	0.5932	0.1616	0.0511	0.015*	0.35
O1B	0.5583 (12)	0.4284 (11)	−0.0007 (7)	0.0124 (7)	0.15
C28B	0.5266 (16)	0.4209 (16)	0.1079 (8)	0.0118 (8)	0.15
H28C	0.4395	0.3790	0.1392	0.014*	0.15
H28D	0.5040	0.5083	0.1299	0.014*	0.15
C29B	0.6701 (15)	0.3396 (16)	0.1373 (10)	0.0126 (6)	0.15
H29C	0.6608	0.2484	0.1609	0.015*	0.15
H29D	0.6966	0.3747	0.1921	0.015*	0.15
C30B	0.7903 (17)	0.3508 (18)	0.0340 (10)	0.0125 (7)	0.15
H30C	0.8286	0.4317	0.0238	0.015*	0.15
H30D	0.8767	0.2754	0.0320	0.015*	0.15
C31B	0.6999 (14)	0.3523 (16)	−0.0446 (11)	0.0125 (7)	0.15
H31C	0.7441	0.3940	−0.1130	0.015*	0.15
H31D	0.6909	0.2638	−0.0506	0.015*	0.15

### Atomic displacement parameters (Å^2^)

**Table:**

	*U*^11^	*U*^22^	*U*^33^	*U*^12^	*U*^13^	*U*^23^
Fe1	0.0091 (3)	0.0097 (3)	0.0089 (3)	−0.00137 (18)	−0.00237 (19)	−0.00117 (18)
N3	0.0125 (11)	0.0108 (10)	0.0129 (10)	−0.0014 (8)	−0.0040 (8)	−0.0004 (8)
N4	0.0117 (10)	0.0141 (11)	0.0160 (11)	−0.0036 (8)	−0.0033 (9)	−0.0013 (9)
N1	0.0110 (10)	0.0118 (10)	0.0120 (10)	−0.0012 (8)	−0.0024 (8)	−0.0013 (8)
N2	0.0109 (10)	0.0112 (10)	0.0107 (10)	−0.0032 (8)	−0.0024 (8)	−0.0011 (8)
C1	0.0125 (12)	0.0152 (12)	0.0113 (12)	−0.0038 (10)	−0.0036 (10)	0.0006 (10)
C2	0.0138 (12)	0.0172 (13)	0.0111 (12)	−0.0024 (10)	−0.0029 (10)	−0.0021 (10)
C3	0.0249 (15)	0.0221 (14)	0.0118 (13)	0.0036 (11)	−0.0059 (11)	−0.0041 (11)
C4	0.0225 (14)	0.0213 (14)	0.0126 (13)	0.0034 (11)	−0.0040 (11)	−0.0061 (11)
C5	0.0117 (12)	0.0162 (13)	0.0130 (12)	−0.0015 (10)	−0.0020 (10)	−0.0043 (10)
C6	0.0111 (12)	0.0122 (12)	0.0152 (12)	−0.0017 (9)	−0.0020 (10)	−0.0041 (10)
C7	0.0101 (12)	0.0105 (12)	0.0156 (12)	−0.0031 (9)	−0.0022 (10)	−0.0018 (10)
C8	0.0168 (13)	0.0104 (12)	0.0176 (13)	−0.0032 (10)	−0.0052 (10)	−0.0014 (10)
C9	0.0167 (13)	0.0115 (12)	0.0158 (13)	−0.0037 (10)	−0.0061 (10)	0.0009 (10)
C10	0.0106 (12)	0.0115 (12)	0.0129 (12)	−0.0039 (9)	−0.0040 (9)	0.0003 (9)
C11	0.0138 (12)	0.0177 (13)	0.0150 (12)	−0.0043 (10)	−0.0031 (10)	−0.0031 (10)
C12	0.0148 (13)	0.0157 (13)	0.0183 (13)	−0.0028 (10)	0.0000 (11)	−0.0038 (10)
C13	0.0141 (13)	0.0183 (13)	0.0207 (14)	−0.0034 (10)	0.0011 (11)	−0.0043 (11)
C14	0.0171 (14)	0.0199 (14)	0.0242 (15)	−0.0095 (11)	−0.0066 (11)	−0.0003 (12)
C15	0.0233 (15)	0.0176 (13)	0.0236 (15)	−0.0058 (11)	−0.0102 (12)	−0.0018 (11)
C16	0.0144 (13)	0.0176 (13)	0.0110 (12)	0.0020 (10)	−0.0027 (10)	−0.0012 (10)
C17	0.0253 (15)	0.0252 (15)	0.0183 (14)	−0.0060 (12)	−0.0050 (12)	0.0027 (12)
C18	0.0369 (18)	0.0284 (17)	0.0189 (15)	−0.0022 (14)	−0.0047 (13)	0.0075 (12)
C19	0.0352 (18)	0.0357 (18)	0.0130 (14)	0.0101 (14)	−0.0093 (13)	0.0001 (12)
C20	0.0250 (16)	0.0354 (18)	0.0175 (14)	0.0070 (13)	−0.0088 (12)	−0.0117 (13)
C21	0.0199 (14)	0.0203 (14)	0.0162 (13)	0.0026 (11)	−0.0042 (11)	−0.0066 (11)
C22	0.0162 (13)	0.0135 (12)	0.0126 (12)	−0.0002 (10)	−0.0070 (10)	−0.0022 (10)
C23	0.0158 (13)	0.0179 (13)	0.0144 (13)	−0.0022 (10)	−0.0049 (10)	−0.0046 (10)
C24	0.0199 (14)	0.0220 (14)	0.0145 (13)	0.0048 (11)	−0.0053 (11)	−0.0073 (11)
C25	0.0300 (16)	0.0152 (13)	0.0182 (14)	0.0046 (11)	−0.0121 (12)	−0.0064 (11)
C26	0.0298 (16)	0.0153 (14)	0.0279 (16)	−0.0061 (12)	−0.0111 (13)	−0.0038 (12)
C27	0.0175 (14)	0.0193 (14)	0.0242 (14)	−0.0042 (11)	−0.0048 (11)	−0.0038 (11)
O1A	0.0166 (15)	0.0189 (15)	0.0051 (13)	−0.0094 (12)	−0.0044 (12)	0.0030 (11)
C29A	0.0171 (15)	0.0180 (15)	0.0047 (13)	−0.0091 (12)	−0.0046 (12)	0.0024 (12)
C28A	0.0169 (15)	0.0187 (15)	0.0049 (14)	−0.0093 (12)	−0.0043 (12)	0.0028 (12)
C30A	0.0174 (15)	0.0176 (15)	0.0042 (13)	−0.0090 (12)	−0.0048 (12)	0.0025 (12)
C31A	0.0170 (15)	0.0185 (15)	0.0050 (13)	−0.0088 (12)	−0.0045 (12)	0.0030 (12)
O1B	0.0180 (16)	0.0178 (16)	0.0042 (14)	−0.0089 (13)	−0.0050 (13)	0.0023 (13)
C28B	0.0168 (16)	0.0179 (16)	0.0039 (15)	−0.0093 (14)	−0.0051 (13)	0.0024 (13)
C29B	0.0170 (15)	0.0185 (15)	0.0048 (13)	−0.0092 (12)	−0.0047 (12)	0.0030 (11)
C30B	0.0174 (15)	0.0181 (15)	0.0045 (14)	−0.0090 (13)	−0.0046 (12)	0.0025 (12)
C31B	0.0177 (16)	0.0182 (16)	0.0044 (15)	−0.0090 (13)	−0.0048 (13)	0.0022 (13)

### Geometric parameters (Å, º)

**Table:**

Fe1—N2	1.992 (2)	C18—C19	1.375 (5)
Fe1—N2^i^	1.992 (2)	C18—H18	0.9500
Fe1—N3	1.994 (2)	C19—C20	1.382 (5)
Fe1—N3^i^	1.994 (2)	C19—H19	0.9500
Fe1—N1^i^	1.998 (2)	C20—C21	1.388 (4)
Fe1—N1	1.998 (2)	C20—H20	0.9500
N3—C11	1.320 (3)	C21—H21	0.9500
N3—C12	1.375 (3)	C22—C23	1.392 (4)
N4—C11	1.346 (3)	C22—C27	1.396 (4)
N4—C13	1.364 (4)	C23—C24	1.389 (4)
N4—C14	1.471 (3)	C23—H23	0.9500
N1—C5	1.381 (3)	C24—C25	1.390 (4)
N1—C2	1.383 (3)	C24—H24	0.9500
N2—C7	1.379 (3)	C25—C26	1.388 (4)
N2—C10	1.382 (3)	C25—H25	0.9500
C1—C10^i^	1.396 (4)	C26—C27	1.390 (4)
C1—C2	1.397 (4)	C26—H26	0.9500
C1—C16	1.494 (3)	C27—H27	0.9500
C2—C3	1.442 (4)	O1A—C28A	1.431 (9)
C3—C4	1.346 (4)	O1A—C31A	1.432 (8)
C3—H3	0.9500	C29A—C28A	1.497 (10)
C4—C5	1.440 (4)	C29A—C30A	1.580 (10)
C4—H4	0.9500	C29A—H29A	0.9900
C5—C6	1.388 (4)	C29A—H29B	0.9900
C6—C7	1.395 (4)	C28A—H28A	0.9900
C6—C22	1.502 (3)	C28A—H28B	0.9900
C7—C8	1.438 (4)	C30A—C31A	1.486 (10)
C8—C9	1.346 (4)	C30A—H30A	0.9900
C8—H8	0.9500	C30A—H30B	0.9900
C9—C10	1.444 (4)	C31A—H31A	0.9900
C9—H9	0.9500	C31A—H31B	0.9900
C10—C1^i^	1.396 (4)	O1B—C28B	1.415 (9)
C11—H11	0.9500	O1B—C31B	1.421 (9)
C12—C13	1.363 (4)	O1B—O1B^ii^	1.65 (2)
C12—H12	0.9500	C28B—C29B	1.505 (10)
C13—H13	0.9500	C28B—H28C	0.9900
C14—C15	1.507 (4)	C28B—H28D	0.9900
C14—H14A	0.97 (4)	C29B—C30B	1.568 (9)
C14—H14B	0.99 (4)	C29B—H29C	0.9900
C15—H15A	0.9800	C29B—H29D	0.9900
C15—H15B	0.9800	C30B—C31B	1.499 (9)
C15—H15C	0.9800	C30B—H30C	0.9900
C16—C17	1.394 (4)	C30B—H30D	0.9900
C16—C21	1.398 (4)	C31B—H31C	0.9900
C17—C18	1.392 (4)	C31B—H31D	0.9900
C17—H17	0.9500		
			
N2—Fe1—N2^i^	180.0	C16—C17—H17	119.7
N2—Fe1—N3	90.27 (9)	C19—C18—C17	120.7 (3)
N2^i^—Fe1—N3	89.72 (9)	C19—C18—H18	119.6
N2—Fe1—N3^i^	89.73 (9)	C17—C18—H18	119.6
N2^i^—Fe1—N3^i^	90.28 (9)	C18—C19—C20	119.5 (3)
N3—Fe1—N3^i^	180.0	C18—C19—H19	120.3
N2—Fe1—N1^i^	89.51 (9)	C20—C19—H19	120.3
N2^i^—Fe1—N1^i^	90.49 (9)	C19—C20—C21	120.2 (3)
N3—Fe1—N1^i^	89.95 (9)	C19—C20—H20	119.9
N3^i^—Fe1—N1^i^	90.05 (9)	C21—C20—H20	119.9
N2—Fe1—N1	90.49 (9)	C20—C21—C16	121.0 (3)
N2^i^—Fe1—N1	89.51 (9)	C20—C21—H21	119.5
N3—Fe1—N1	90.05 (9)	C16—C21—H21	119.5
N3^i^—Fe1—N1	89.95 (9)	C23—C22—C27	118.9 (2)
N1^i^—Fe1—N1	180.0	C23—C22—C6	120.9 (2)
C11—N3—C12	105.6 (2)	C27—C22—C6	120.2 (2)
C11—N3—Fe1	125.62 (19)	C24—C23—C22	120.4 (3)
C12—N3—Fe1	128.74 (18)	C24—C23—H23	119.8
C11—N4—C13	107.0 (2)	C22—C23—H23	119.8
C11—N4—C14	127.4 (2)	C23—C24—C25	120.4 (3)
C13—N4—C14	125.5 (2)	C23—C24—H24	119.8
C5—N1—C2	105.2 (2)	C25—C24—H24	119.8
C5—N1—Fe1	127.09 (17)	C26—C25—C24	119.6 (3)
C2—N1—Fe1	127.71 (18)	C26—C25—H25	120.2
C7—N2—C10	105.1 (2)	C24—C25—H25	120.2
C7—N2—Fe1	126.70 (17)	C25—C26—C27	120.0 (3)
C10—N2—Fe1	128.22 (17)	C25—C26—H26	120.0
C10^i^—C1—C2	123.5 (2)	C27—C26—H26	120.0
C10^i^—C1—C16	118.7 (2)	C26—C27—C22	120.7 (3)
C2—C1—C16	117.8 (2)	C26—C27—H27	119.7
N1—C2—C1	125.7 (2)	C22—C27—H27	119.7
N1—C2—C3	110.1 (2)	C28A—O1A—C31A	109.1 (5)
C1—C2—C3	124.3 (2)	C28A—C29A—C30A	102.9 (6)
C4—C3—C2	107.3 (2)	C28A—C29A—H29A	111.2
C4—C3—H3	126.3	C30A—C29A—H29A	111.2
C2—C3—H3	126.3	C28A—C29A—H29B	111.2
C3—C4—C5	107.0 (2)	C30A—C29A—H29B	111.2
C3—C4—H4	126.5	H29A—C29A—H29B	109.1
C5—C4—H4	126.5	O1A—C28A—C29A	107.4 (6)
N1—C5—C6	125.6 (2)	O1A—C28A—H28A	110.2
N1—C5—C4	110.4 (2)	C29A—C28A—H28A	110.2
C6—C5—C4	124.0 (2)	O1A—C28A—H28B	110.2
C5—C6—C7	123.9 (2)	C29A—C28A—H28B	110.2
C5—C6—C22	118.7 (2)	H28A—C28A—H28B	108.5
C7—C6—C22	117.3 (2)	C31A—C30A—C29A	100.8 (5)
N2—C7—C6	126.2 (2)	C31A—C30A—H30A	111.6
N2—C7—C8	110.5 (2)	C29A—C30A—H30A	111.6
C6—C7—C8	123.3 (2)	C31A—C30A—H30B	111.6
C9—C8—C7	107.3 (2)	C29A—C30A—H30B	111.6
C9—C8—H8	126.4	H30A—C30A—H30B	109.4
C7—C8—H8	126.4	O1A—C31A—C30A	109.3 (6)
C8—C9—C10	106.9 (2)	O1A—C31A—H31A	109.8
C8—C9—H9	126.6	C30A—C31A—H31A	109.8
C10—C9—H9	126.6	O1A—C31A—H31B	109.8
N2—C10—C1^i^	125.3 (2)	C30A—C31A—H31B	109.8
N2—C10—C9	110.3 (2)	H31A—C31A—H31B	108.3
C1^i^—C10—C9	124.3 (2)	C28B—O1B—C31B	115.1 (11)
N3—C11—N4	111.6 (2)	C28B—O1B—O1B^ii^	86.3 (9)
N3—C11—H11	124.2	C31B—O1B—O1B^ii^	147.8 (13)
N4—C11—H11	124.2	O1B—C28B—C29B	103.8 (11)
C13—C12—N3	109.2 (2)	O1B—C28B—H28C	111.0
C13—C12—H12	125.4	C29B—C28B—H28C	111.0
N3—C12—H12	125.4	O1B—C28B—H28D	111.0
C12—C13—N4	106.6 (2)	C29B—C28B—H28D	111.0
C12—C13—H13	126.7	H28C—C28B—H28D	109.0
N4—C13—H13	126.7	C28B—C29B—C30B	102.7 (11)
N4—C14—C15	112.8 (2)	C28B—C29B—H29C	111.2
N4—C14—H14A	106 (2)	C30B—C29B—H29C	111.2
C15—C14—H14A	113 (2)	C28B—C29B—H29D	111.2
N4—C14—H14B	106 (2)	C30B—C29B—H29D	111.2
C15—C14—H14B	114 (2)	H29C—C29B—H29D	109.1
H14A—C14—H14B	104 (3)	C31B—C30B—C29B	102.1 (11)
C14—C15—H15A	109.5	C31B—C30B—H30C	111.4
C14—C15—H15B	109.5	C29B—C30B—H30C	111.4
H15A—C15—H15B	109.5	C31B—C30B—H30D	111.4
C14—C15—H15C	109.5	C29B—C30B—H30D	111.4
H15A—C15—H15C	109.5	H30C—C30B—H30D	109.2
H15B—C15—H15C	109.5	O1B—C31B—C30B	100.4 (11)
C17—C16—C21	117.9 (3)	O1B—C31B—H31C	111.7
C17—C16—C1	121.7 (3)	C30B—C31B—H31C	111.7
C21—C16—C1	120.4 (2)	O1B—C31B—H31D	111.7
C18—C17—C16	120.6 (3)	C30B—C31B—H31D	111.7
C18—C17—H17	119.7	H31C—C31B—H31D	109.5
			
C5—N1—C2—C1	177.7 (3)	Fe1—N3—C12—C13	−178.97 (19)
Fe1—N1—C2—C1	−2.8 (4)	N3—C12—C13—N4	−0.1 (3)
C5—N1—C2—C3	−2.0 (3)	C11—N4—C13—C12	−0.3 (3)
Fe1—N1—C2—C3	177.45 (19)	C14—N4—C13—C12	−176.0 (2)
C10^i^—C1—C2—N1	3.6 (4)	C11—N4—C14—C15	24.6 (4)
C16—C1—C2—N1	−175.7 (2)	C13—N4—C14—C15	−160.6 (3)
C10^i^—C1—C2—C3	−176.7 (3)	C10^i^—C1—C16—C17	59.0 (4)
C16—C1—C2—C3	4.0 (4)	C2—C1—C16—C17	−121.7 (3)
N1—C2—C3—C4	2.0 (3)	C10^i^—C1—C16—C21	−120.9 (3)
C1—C2—C3—C4	−177.8 (3)	C2—C1—C16—C21	58.4 (4)
C2—C3—C4—C5	−1.0 (3)	C21—C16—C17—C18	0.4 (4)
C2—N1—C5—C6	−178.1 (3)	C1—C16—C17—C18	−179.5 (3)
Fe1—N1—C5—C6	2.4 (4)	C16—C17—C18—C19	−1.2 (5)
C2—N1—C5—C4	1.4 (3)	C17—C18—C19—C20	1.1 (5)
Fe1—N1—C5—C4	−178.10 (18)	C18—C19—C20—C21	−0.2 (5)
C3—C4—C5—N1	−0.2 (3)	C19—C20—C21—C16	−0.6 (4)
C3—C4—C5—C6	179.3 (3)	C17—C16—C21—C20	0.5 (4)
N1—C5—C6—C7	−3.2 (4)	C1—C16—C21—C20	−179.6 (3)
C4—C5—C6—C7	177.4 (3)	C5—C6—C22—C23	82.2 (3)
N1—C5—C6—C22	176.5 (2)	C7—C6—C22—C23	−98.0 (3)
C4—C5—C6—C22	−2.8 (4)	C5—C6—C22—C27	−99.3 (3)
C10—N2—C7—C6	−178.6 (2)	C7—C6—C22—C27	80.5 (3)
Fe1—N2—C7—C6	0.0 (4)	C27—C22—C23—C24	−0.2 (4)
C10—N2—C7—C8	−0.7 (3)	C6—C22—C23—C24	178.3 (2)
Fe1—N2—C7—C8	177.96 (17)	C22—C23—C24—C25	0.5 (4)
C5—C6—C7—N2	1.9 (4)	C23—C24—C25—C26	−0.7 (4)
C22—C6—C7—N2	−177.8 (2)	C24—C25—C26—C27	0.5 (4)
C5—C6—C7—C8	−175.8 (2)	C25—C26—C27—C22	−0.2 (4)
C22—C6—C7—C8	4.5 (4)	C23—C22—C27—C26	0.0 (4)
N2—C7—C8—C9	−0.2 (3)	C6—C22—C27—C26	−178.5 (3)
C6—C7—C8—C9	177.8 (2)	C31A—O1A—C28A—C29A	10.8 (8)
C7—C8—C9—C10	0.9 (3)	C30A—C29A—C28A—O1A	−26.6 (8)
C7—N2—C10—C1^i^	−179.2 (2)	C28A—C29A—C30A—C31A	31.6 (7)
Fe1—N2—C10—C1^i^	2.3 (4)	C28A—O1A—C31A—C30A	11.1 (8)
C7—N2—C10—C9	1.2 (3)	C29A—C30A—C31A—O1A	−26.5 (8)
Fe1—N2—C10—C9	−177.34 (17)	C31B—O1B—C28B—C29B	5.3 (17)
C8—C9—C10—N2	−1.4 (3)	O1B^ii^—O1B—C28B—C29B	−149.7 (13)
C8—C9—C10—C1^i^	179.0 (2)	O1B—C28B—C29B—C30B	19.5 (16)
C12—N3—C11—N4	−0.5 (3)	C28B—C29B—C30B—C31B	−36.3 (16)
Fe1—N3—C11—N4	178.81 (17)	C28B—O1B—C31B—C30B	−28.6 (17)
C13—N4—C11—N3	0.5 (3)	O1B^ii^—O1B—C31B—C30B	99 (2)
C14—N4—C11—N3	176.1 (2)	C29B—C30B—C31B—O1B	38.1 (16)
C11—N3—C12—C13	0.4 (3)		

Symmetry codes: (i) −*x*, −*y*+1, −*z*+1; (ii) −*x*+1, −*y*+1, −*z*.

### Hydrogen-bond geometry (Å, º)

Cg is the centroid of the N2/C7–C10 ring.

**Table:**

*D*—H···*A*	*D*—H	H···*A*	*D*···*A*	*D*—H···*A*
C14—H14*B*···*Cg*^iii^	0.99 (4)	2.69 (4)	3.437 (3)	133 (2)

Symmetry code: (iii) −*x*+1, −*y*+1, −*z*+1.
